# Supplementary material for: Out-of-equilibrium microcompartments for the bottom-up integration of metabolic functions
Source: Nat Commun. 2018 Jun 19;9:2391. doi: 10.1038/s41467-018-04825-1 (PMC6008305; doi:10.1038/s41467-018-04825-1)
Supplement: Supplementary file 1 — Supplementary Information [file 41467_2018_4825_MOESM1_ESM.pdf]

Supplementary Information

Out-of-equilibrium microcompartments for the bottom-up integration of metabolic functions

Beneyton *et al.*

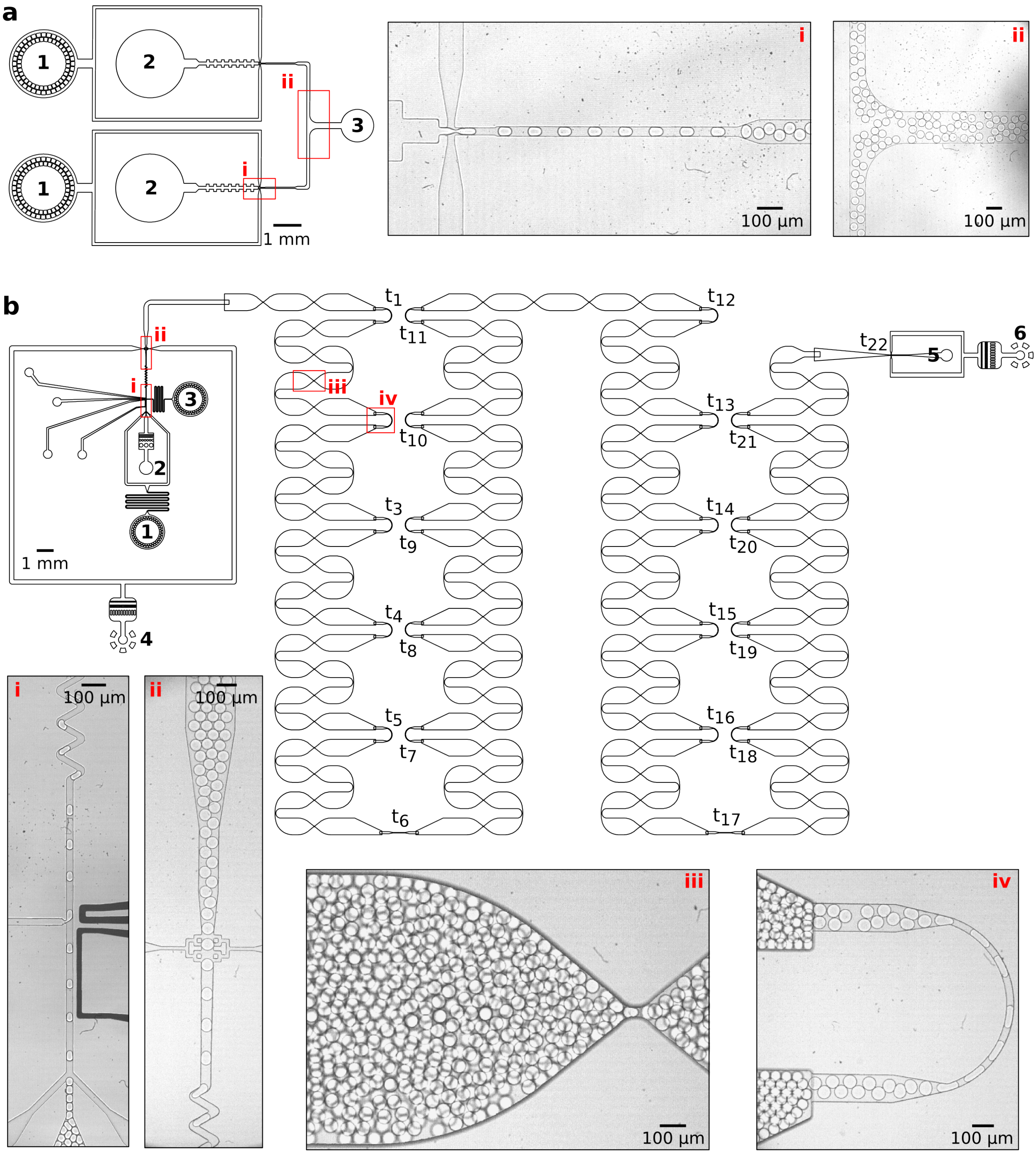

Supplementary Fig. 1: Microfluidic workflow 1. (a) Dropmaker. 30 pL w/o droplets are generated by the parallelized flow-focusing of two aqueous streams (2) with two streams of fluorinated oil containing surfactant (1). Two droplets populations are collected together in a glass vial (3). The nozzle dimensions are 30x30x20 μm. (b) Kinetics module. 30 pL w/o droplets are reloaded (2) and spaced with two streams of fluorinated oil containing surfactant (1). Droplets are picoinjected with an aqueous phase (3) by applying AC field (20kHz; 100 V_{pp}). Oil is extracted (4) and packed emulsion is incubated on-chip in delay-lines (iii). Fluorescence is measured in detection loops (iv). When exiting the delay line, the droplets are spaced again with fluorinated oil (5) and flow out the chip (6). The incubation line is 70 μm-depth while the picoinjection part and the detection loops are 20 μm-depth.

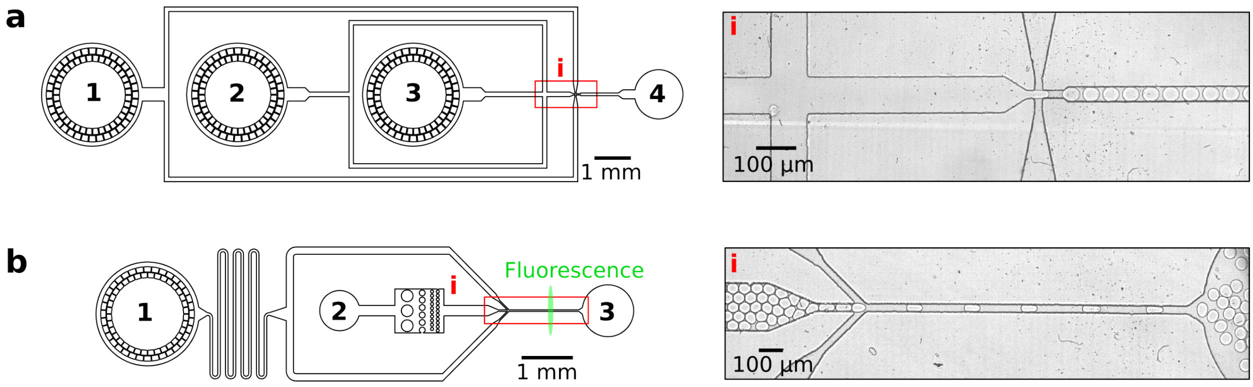

Supplementary Fig. 2: Microfluidic workflow 2. (a) Dropmaker. 30 pL w/o droplets are generated by flow-focusing of two co-flown aqueous streams (2 and 3) with two streams of fluorinated oil containing surfactant (1). Droplets are collected in a glass vial (4). The nozzle dimensions are 30x25x20 μm. (b) Simple reinjection device. Droplets are co-flown with two streams of fluorinated oil and droplet fluorescence is measured. The detection channel is 30x20 μm.

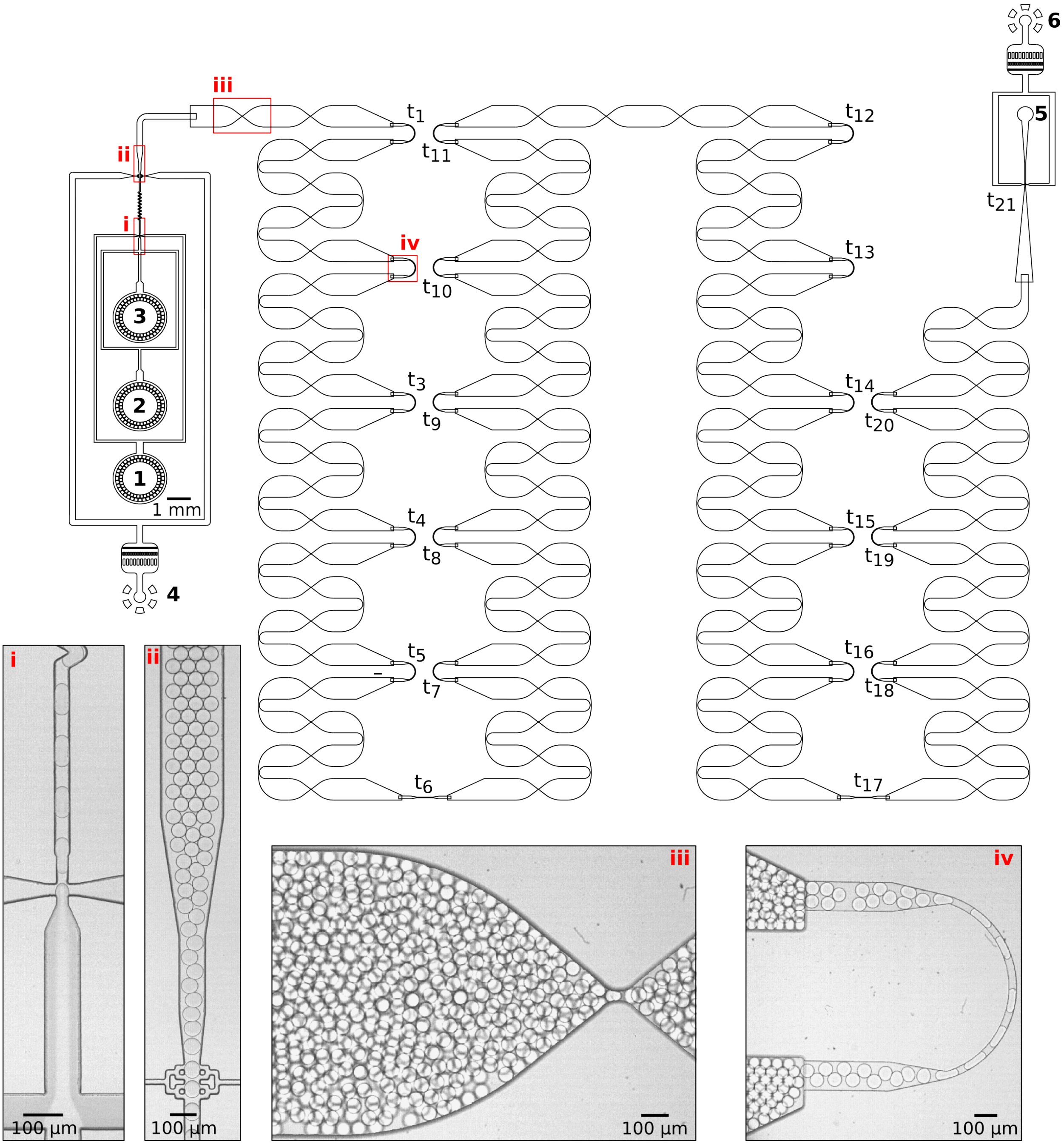

Supplementary Fig. 3: Microfluidic workflow 3. 90 pL w/o droplets are generated by flow-focusing of two co-flown aqueous streams (2 and 3) with two streams of fluorinated oil containing surfactant (1). The nozzle dimensions are 30x30x20 μm. Oil is extracted (4) and packed emulsion is incubated on-chip in delay-lines (iii). Fluorescence is measured in detection loops (iv). When exiting the delay line, the droplets are spaced again with fluorinated oil (5) and flow out the chip (6). The incubation line is 70 μm-depth while the dropmaker part and the detection loops are 20 μm-depth.

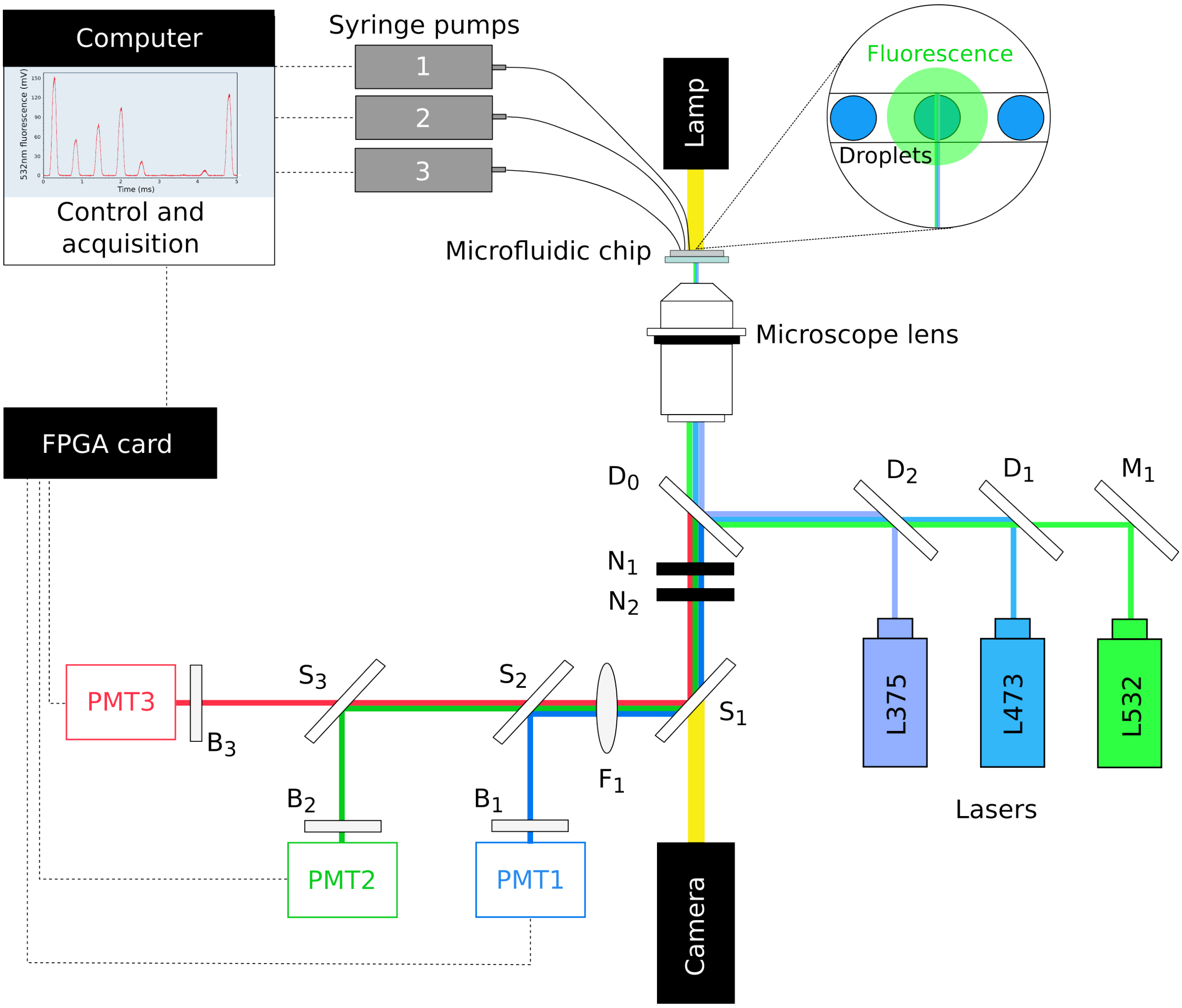

Supplementary Fig. 4: Optical setup. The three lasers (375 nm with 20 mW, Omicron; 473 nm with 250 mW, Cobolt; 532 nm with 25 mW, Cobolt Samba) are focused with an objective (40x; Olympus) into the microfluidic channels using a set of mirrors and dichroics (M_1 = ND10A, Thorlabs; D_1 = F38-M03, AHF; D_2 = F38-M01, AHF; D_0 = F73-049, AHF). The microfluidic chip is mounted on the x-y stage of an inverted microscope (IX71, Olympus). Droplets are observed with a high-speed camera (v210, Phantom) using a white LED (Olympus) from above. The LED is turned off for data acquisition of the fluorescence signal of the droplets. The dyes in the droplets are excited by the lasers and the fluorescent light is directed through several notches and filters and split with dichroics (N_1 = F40-473 (AHF), N_2 = F40-532 (AHF), S_1 = F21-002 (80R/20T, AHF), F_1 = 30mm lens, S_2 = F38-458 (AHF), B_1 = F39-438 (AHF), S_3 = F33-563 (AHF), B_2 = F37-524 (AHF), B_3 = F37-580 (AHF)) to be recorded by three photomultipliers (PMTs, H9656-20; Hamamatsu). PMTs respective windows are: 424-453 nm (PMT1), 497-545 nm (PMT2) and 565-595 nm (PMT3). Data acquisition (DAQ) and control were performed by a DAQ card (National Instruments) executing a program written in LabView (National Instruments). The data acquisition rate for the system was 200 kHz.

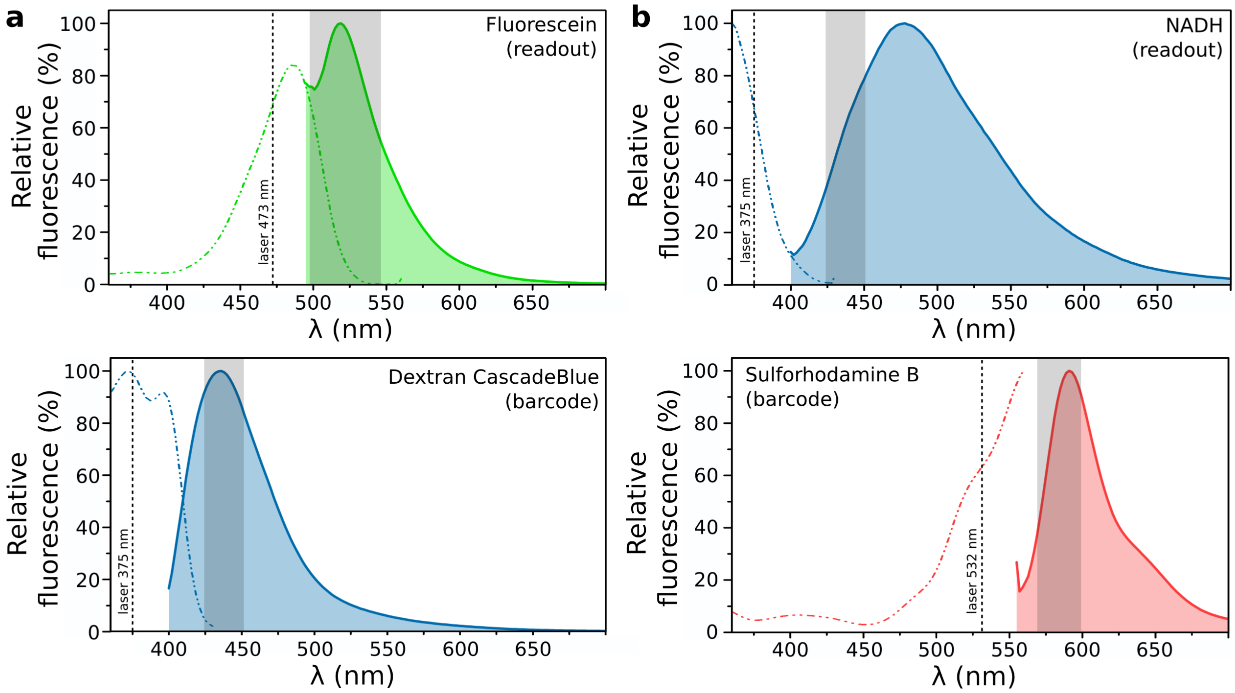

Supplementary Fig. 5: Fluorescence spectrum of readout/barcode fluorophore couples. (a) Fluorescein/Dextran CascadeBlue. Fluorescein: λ_{ex} = 473 nm and λ_{em} = 525 nm. Dextran CascadeBlue: λ_{ex} = 375 nm and λ_{em} = 450 nm. (b) NADH/Sulforhodamine B. NADH: λ_{ex} = 375 nm and λ_{em} = 450 nm. Sulforhodamine B: λ_{ex} = 532 nm and λ_{em} = 585 nm. The features of the microfluidic optical set up are shown on each spectra (laser wavelengths, emission windows of the PMTs (grey)). All spectrum are recorded in NaOH-Tricine buffer (100 mM, pH 8.0) containing 5 mM $MgCl_2$ at room temperature.

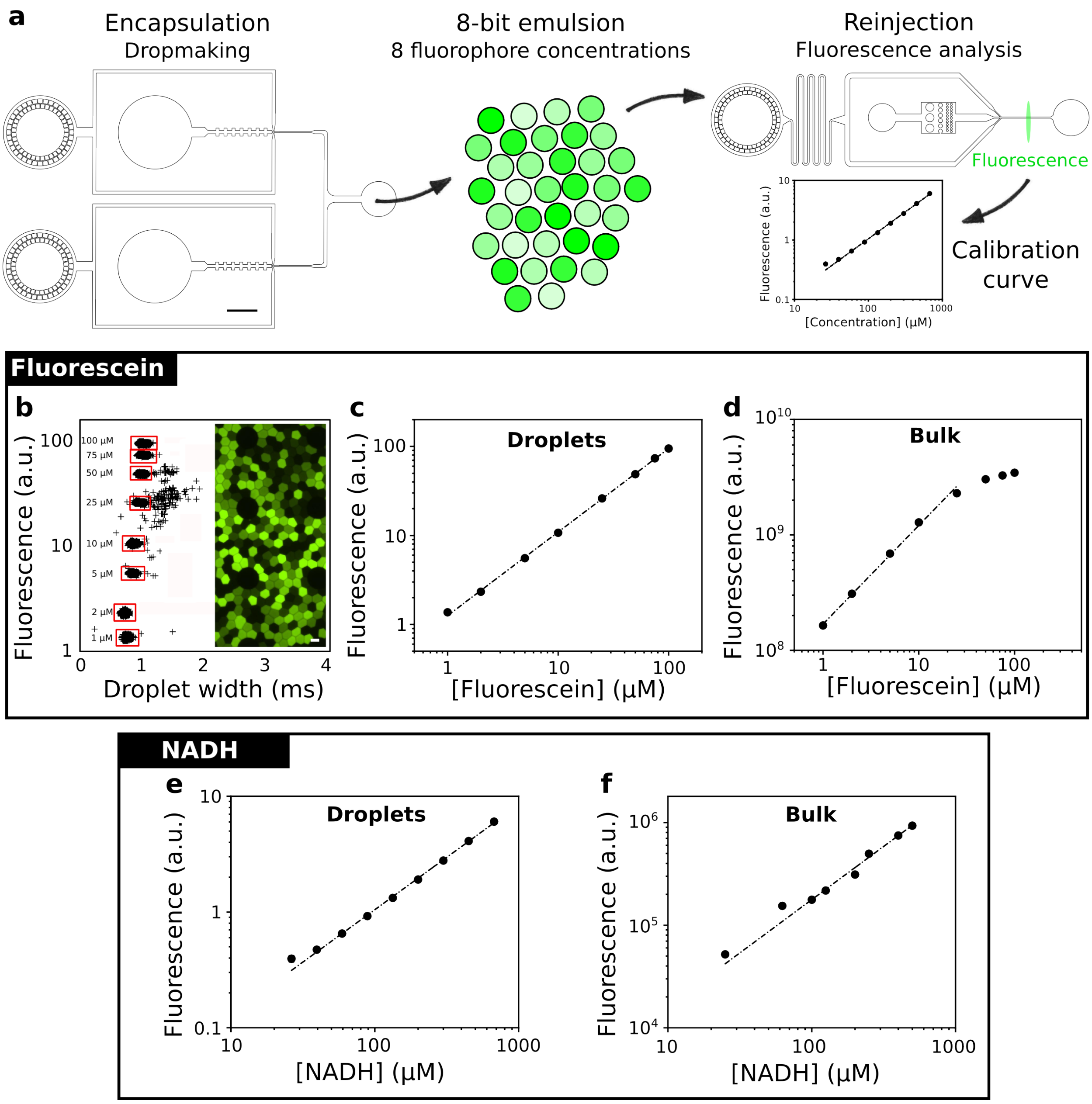

Supplementary Fig. 6: Fluorescence calibration curves. **(a)** Graphic view of the calibration workflow. **(b-c)** Fluorescein in droplets. **(b)** 2D histogram of fluorescence versus droplet width for a 8-bit emulsion composed of 8 different fluorescein concentrations (1, 2, 5, 10, 25, 50, 75 and 100 μM). Epifluorescence picture of the 8-bit emulsion. Scale bar is 30 μm. **(c)** Fluorescence versus fluorescein concentration plot. **(d)** Fluorescein in bulk. Fluorescence versus fluorescein concentration plot from values recorded in a 384-well plate (λ_{ex} = 473 nm; λ_{em} = 520 nm). **(e)** NADH in droplets. Fluorescence versus NADH concentration plot for a 9-bit emulsion composed of 9 different NADH concentrations (26, 39, 59, 89, 133, 200, 300, 450 and 675 μM). **(f)** NADH in bulk. Fluorescence versus NADH concentration plot from values recorded in a 384-well plate (λ_{ex} = 375 nm; λ_{em} = 450 nm). Calibrations are performed in NaOH-Tricine buffer (100 mM, pH 8.0) with $MgCl_2$ 5 mM. Error bars are defined as s.d. (N = 3 in bulk, N = 5 000 in droplets).

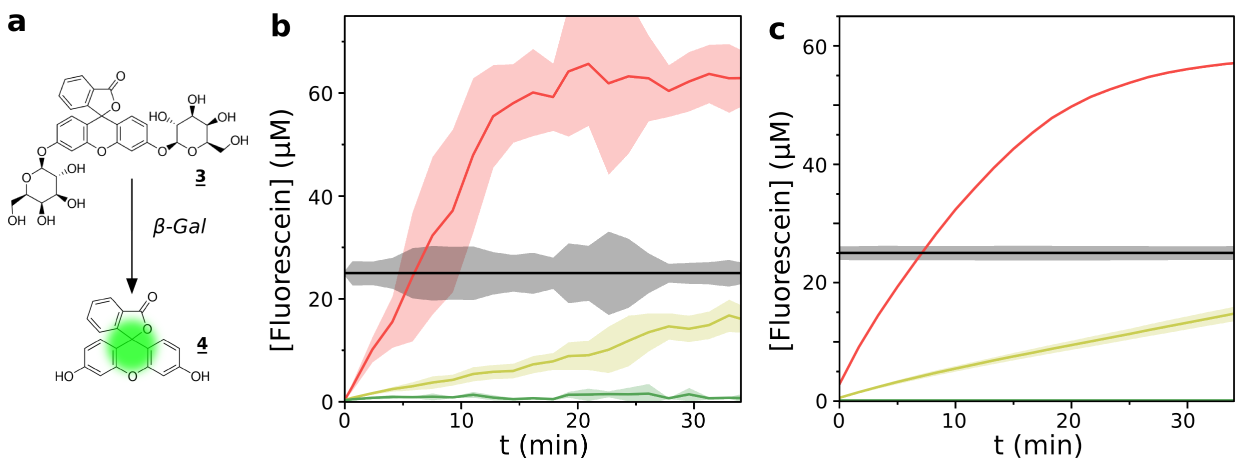

Supplementary Fig. 7: β-galactosidase activity. **(a)** Reaction scheme. β-galactosidase (β-Gal) catalyzes the hydrolysis of fluorescein di(β-D-galactopyranoside) (FDG) into galactose and fluorescein. **(b)** Reaction in 30 pL droplets. Fluorescein concentration versus time (t) of 30 pL w/o droplets containing β-Gal 0, 2.5 and 10 U mL^{-1} (green, yellow and red respectively) or fluorescein 25 μM internal reference (black) after injection of FDG substrate (50 μM). Droplets are barcoded with Dextran-Cascade Blue (5, 10, 20 and 40 μM). Error bars are defined as s.d. (N = 2 000). **(c)** Reaction in bulk. Fluorescein concentration versus time (t) recorded in a 384-well plate (λ_{ex} = 473 nm; λ_{em} = 520 nm) within 50 μL under the same condition as (b). Error bars are defined as s.d. (N = 3). Reactions are performed in NaOH-Tricine buffer (100 mM, pH 8.0) with $MgCl_2$ 5 mM.

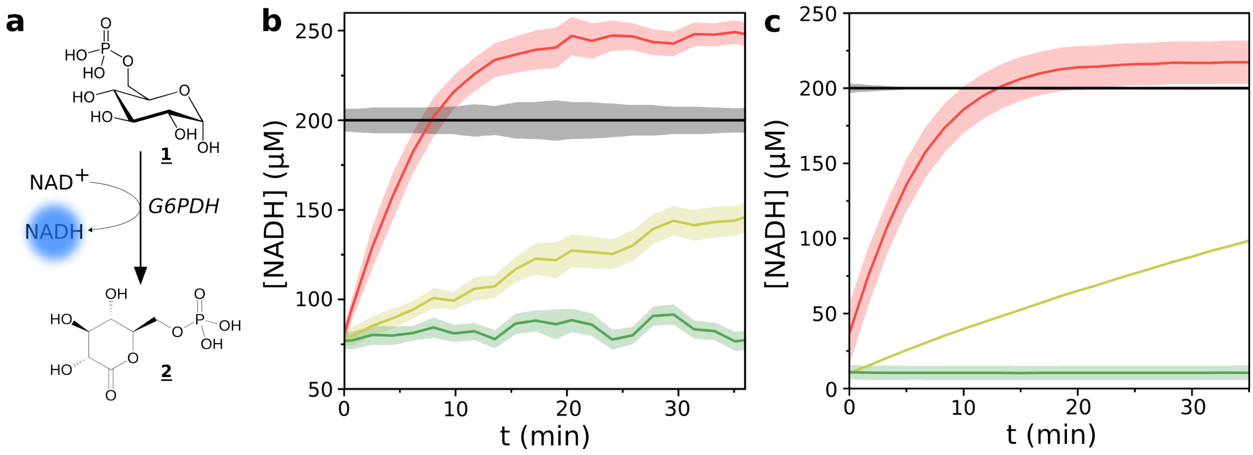

Supplementary Fig. 8: Glucose-6-phosphate dehydrogenase activity. **(a)** Reaction scheme. Glucose-6-phosphate dehydrogenase (G6PDH) oxidizes D-glucose-6-phosphate 1 (G6P) into 6-phospho-D-glucono-1,5-lactone 2 (GLP) with the concomitant reduction of NAD^+ into NADH. **(b)** Reaction in 30 pL droplets. NADH concentration versus time (t) of 30 pL w/o droplets containing NAD^+ (250 μM) and G6PDH 0 or 0.01 or 0.08 U mL^{-1} (green, yellow and red respectively) or NADH 200 μM internal reference (black) after injection of G6P substrate (1 mM). Droplets are barcoded with sulforhodamine B (30, 60, 90 and 120 μM). Error bars are defined as s.d. (N = 2 000). **(c)** Reaction in bulk. NADH concentration versus time (t) recorded in a 384-well plate (λ_{ex} = 375 nm; λ_{em} = 450 nm) within 50 μL under the same condition as (b). Error bars are defined as s.d. (N = 3). Reactions are performed in NaOH-Tricine buffer (100 mM, pH 8.0) with $MgCl_2$ 5 mM.

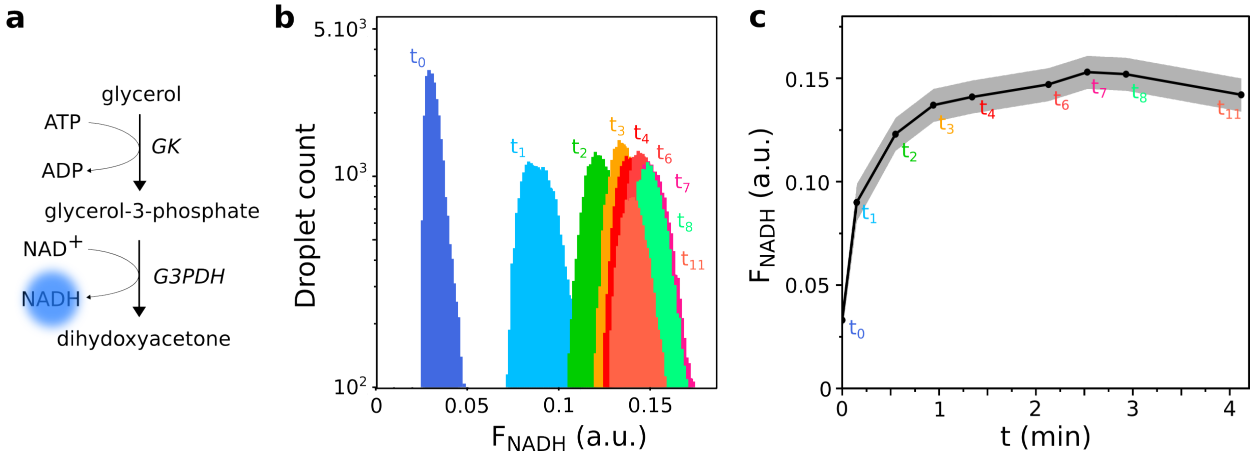

Supplementary Fig. 9: Glycerol-3-phosphate dehydrogenase activity. **(a)** Reaction scheme. Glycerol kinase (GK) catalyzes the transfer of a phosphate from ATP to glycerol to form glycerol-3-phosphate (G3P) and ADP. Glycerol-3-phosphate dehydrogenase (G3PDH) oxidizes G3P into dihydroxyacetone phosphate with the concomitant reduction of NAD^+ into NADH. **(b-c)** Reaction in 90 pL droplets. **(b)** 1D histograms of NADH fluorescence at different incubation time for droplets containing GK (10 $U.mL^{-1}$), G3PDH (10 U mL^{-1}), ATP (10 mM), NAD^+ (5 mM), Glycerol (8 mM) and $MgCl_2$ (100 mM) in NaOH-Glycine buffer (100 mM; pH9.0). **(c)** NADH fluorescence versus time (t) of droplets with the same composition as (b). Error bars are defined as s.d. (N = 2 000).

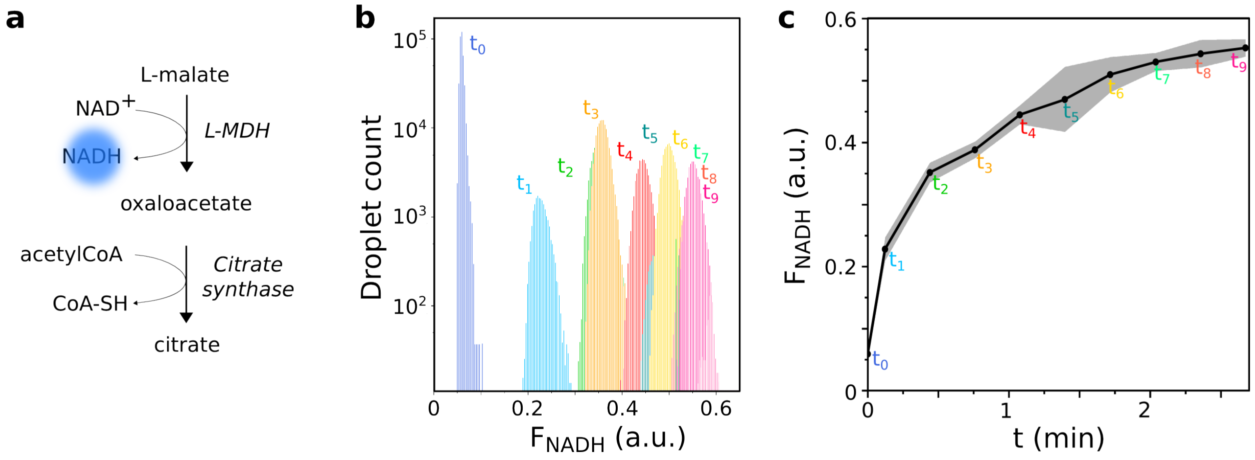

Supplementary Fig. 10: L-malate dehydrogenase activity. **(a)** Reaction scheme. L-malate dehydrogenase (L-MDH) oxidizes L-malate into oxaloacetate with the concomitant reduction of NAD^+ into NADH. Citrate synthase then catalyzes the condensation reaction between acetyl coenzyme A (acetylCoA) and oxaloacetate to form citrate. **(b-c)** Reaction in 90 pL droplets. **(b)** 1D histograms of NADH fluorescence at different incubation time for droplets containing LMDH (5 U mL^{-1}), citrate synthase (5 U mL^{-1}), acetylCoA (0.2 mM), NAD^+ (250 μM) and L-malate (0.3 mM) in KOH-Tricine buffer (100 mM; pH 8.0). **(c)** NADH fluorescence versus time (t) of droplets with the same composition as (b). Error bars are defined as s.d. (N = 2 000).

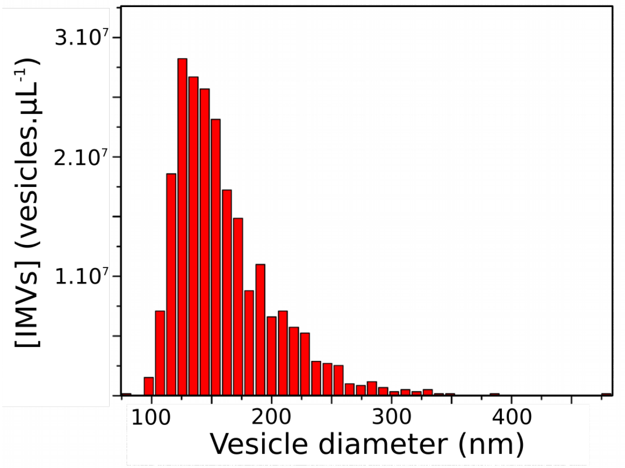

Supplementary Fig. 11: Inverted Membrane Vesicles size distribution. IMVs size versus IMVs concentration. Concentration and size of the vesicles were determined using tunable resistive pulse sensing (TRPS).

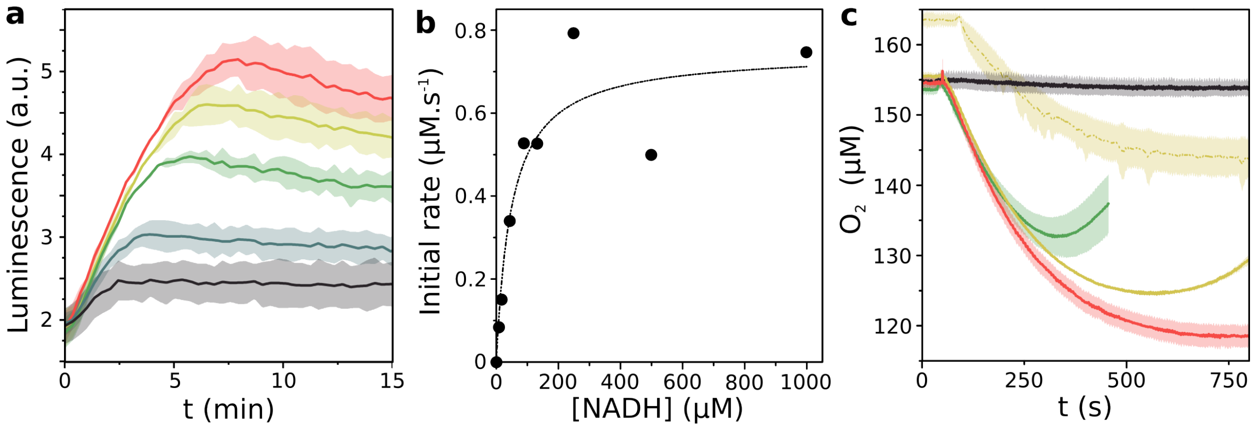

Supplementary Fig. 12: NADH-driven ATP synthase activity and oxygen consumption of IMVs. (a) ATP production is monitored using the luciferin/luciferase assay (Supplementary Note 3). Luminescence signal versus time (t) of solutions containing 5.3×10^9 vesicles per mL after addition of increasing amounts of NADH: 18 (black), 44 (blue), 89 (green), 133 (yellow) and 178 μM (red). (b) Initial rates of NADH oxidation versus NADH concentrations for solutions containing 5.3×10^9 vesicles per mL. (c) Oxygen consumption of IMVs was determined using an oxygraph (Supplementary Note 3). Oxygen concentration versus time (t) for solution containing 4.4×10^9 vesicles per mL and 0 (black), 250 (green), 500 (yellow) or 1000 μM (red) NADH. The dotted yellow curve is in the case of 500 μM NADH and twice less IMVs (2.2×10^9 vesicles per mL). Error bars are defined as s.d. (N = 3).

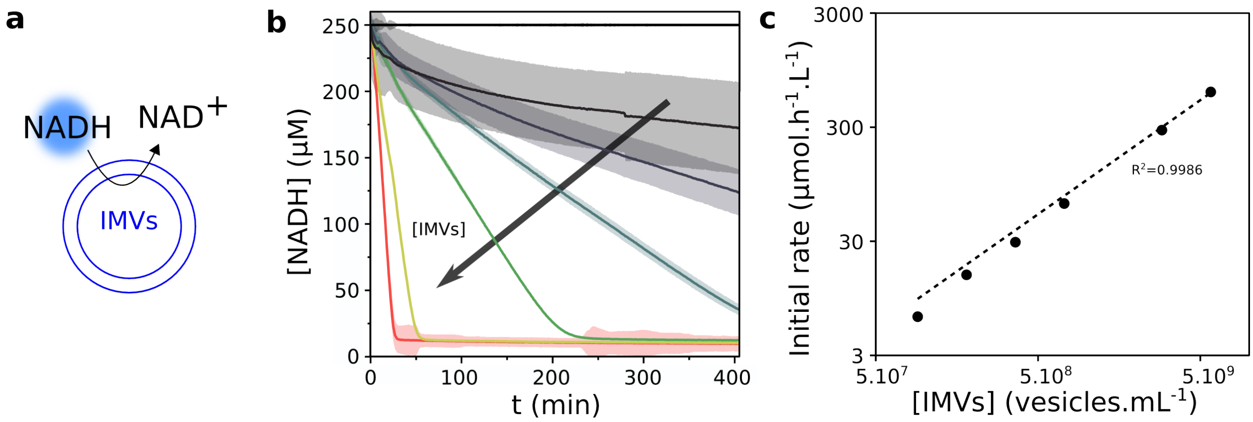

Supplementary Fig. 13: NADH oxidation activity of IMVs. **(a)** Reaction scheme. NADH is oxidized into NAD^+ by the NADH dehydrogenase activity of the respiratory chain of the IMVs. **(b)** Kinetics. NADH concentration versus time (t) for increasing IMVs concentrations (58×10^8, 29×10^8, 7.2×10^8, 3.6×10^8, 1.8×10^8, 0.9×10^8 or 0 vesicles per mL) suspended in NaOH-Tricine buffer (100 mM, pH 8.0) containing $MgCl_2$ (5 mM) and NADH (250 μM). Recorded in 45 μL in a 384-well plate (λ_{ex} = 375 nm; λ_{em} = 450 nm).**(c)** Linear correlation between IMVs concentration and initial reaction rate. Reactions are performed in NaOH-Tricine buffer (100 mM, pH 8.0) with $MgCl_2$ 5 mM. Error bars are defined as s.d. (N = 3).

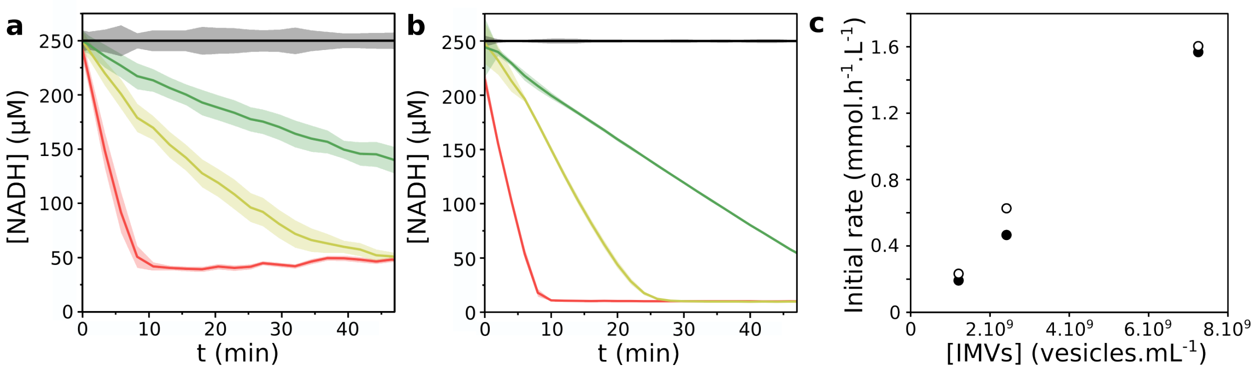

Supplementary Fig. 14: Encapsulated Inverted Membrane Vesicles activity. **(a)** Kinetics in 30 pL droplets. NADH concentration versus time (t) of 30 pL w/o droplets containing IMVs (0, 55, 110 and 330 vesicles per droplet (black, green, yellow and red respectively)) after injection of NADH (250 μM). Error bars are defined as s.d. (N = 2 000). **(b)** Kinetics in bulk. NADH concentration versus time (t) recorded in a 384-well plate (λ_{ex} = 375 nm; λ_{em} = 450 nm) within 50 μL under the same condition as (b). Error bars are defined as s.d. (N = 3). **(c)** Linear correlation between IMVs concentration and initial reaction rate from droplets (black circles) or bulk (open circles) experiments. Reactions are performed in NaOH-Tricine buffer (100 mM, pH 8.0) with $MgCl_2$ 5 mM.

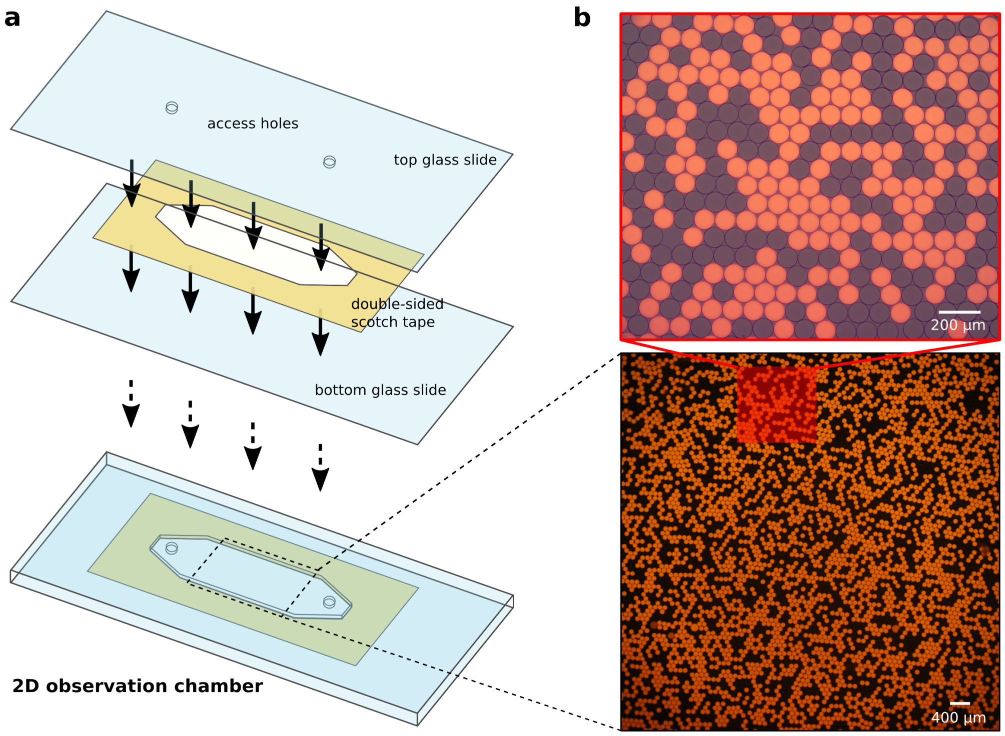

Supplementary Fig. 15: 2D observation chamber. **(a)** Graphic view of the chamber assembly. The assembly protocol is described in Supplementary Note 3. **(b)** Red fluorescence images of a 2-bit emulsion immobilized in the 2D observation chamber. The 300 pL droplets contain either 0 or 100 μM of sulforhodamine B.

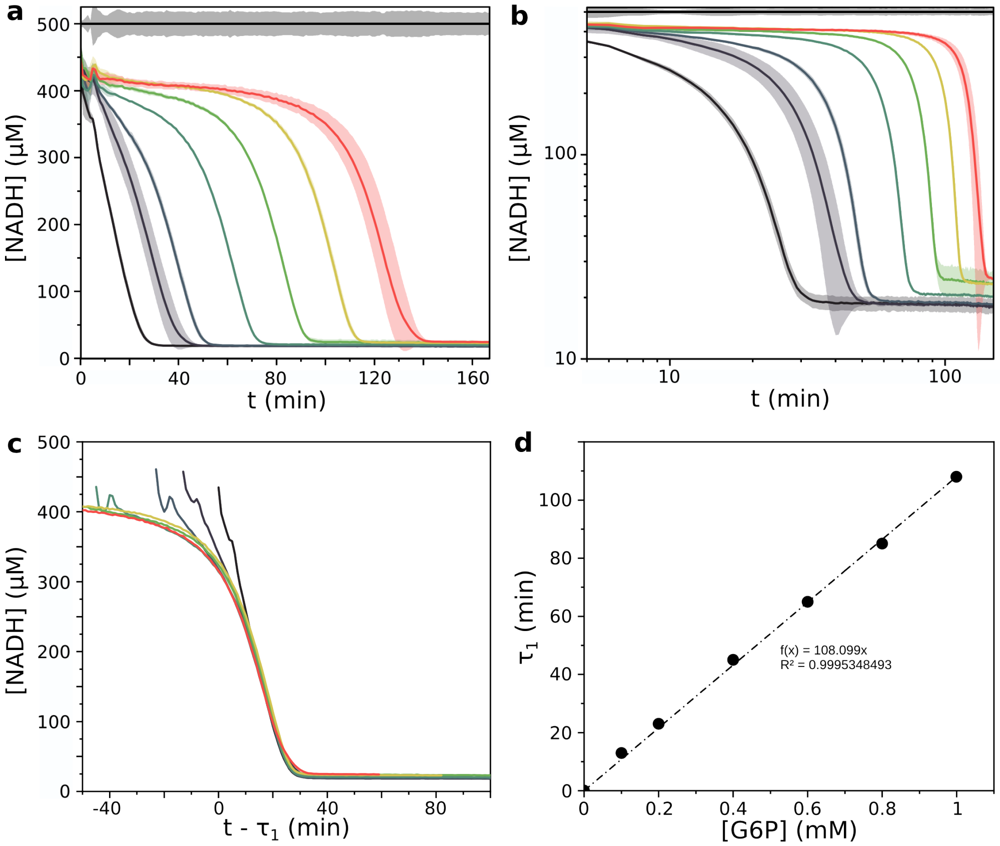

Supplementary Fig. 16: G6P dependency. **(a-b)** NADH concentration versus time (t) of solutions containing NADH (500 μM), IMVs (1.3×10^9 vesicles per mL), G6PDH (0.1 U mL^{-1}) and increasing concentrations of G6P (0, 0.1, 0.2, 0.4, 0.6, 0.8 and 1 mM). Control solution without IMVs in black. **(c)** Collapsing of all the decay data when shifted by respective plateau time τ_1. **(d)** Dependency of the lifetime of the reaction (τ_1) towards G6P concentration. Reactions are performed in NaOH-Tricine buffer (100 mM, pH 8.0) with $MgCl_2$ 5 mM. Error bars are defined as s.d. (N = 3).

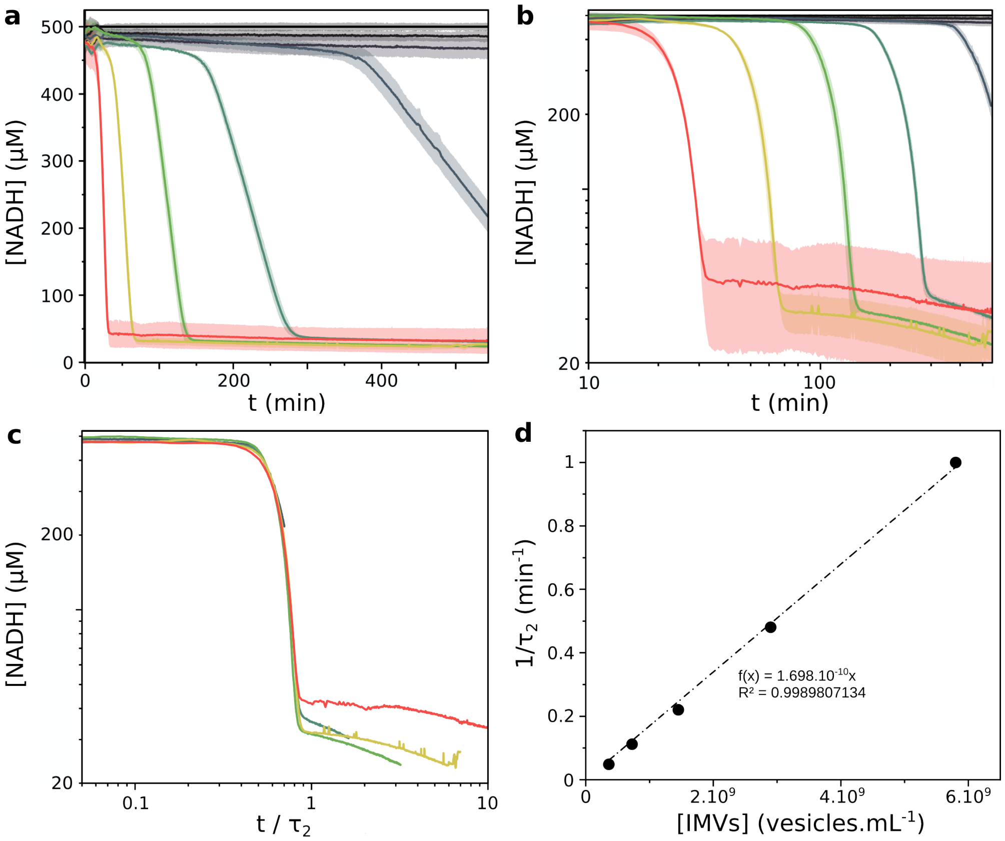

Supplementary Fig. 17: IMVs dependency. **(a-b)** NADH concentration versus time (t) of solutions containing NADH (500 μM), G6P (0.8 mM), G6PDH (0.5 U mL^{-1}) and decreasing concentrations of IMVs (0, 9×10^7, 1.8×10^8, 3.6×10^8, 7.2×10^8, 14.5×10^8, 2.9×10^9 and 5.8×10^9 vesicles per mL). **(c)** Collapsing of all the data when rescaled by a single respective time-scale τ_2. **(d)** Dependency of the lifetime of the reaction (τ_2) towards IMVs concentration. Reactions are performed in NaOH-Tricine buffer (100 mM, pH 8.0) with $MgCl_2$ 5 mM. Error bars are defined as s.d. (N = 3).

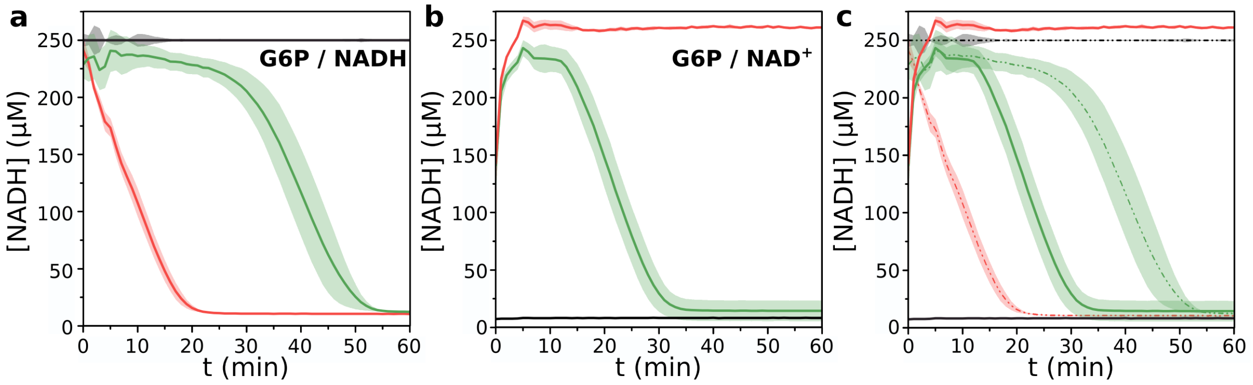

Supplementary Fig. 18: Self-sustained reaction starting form NAD^+. **(a)** G6P/NADH couple. NADH concentration versus time (t) of solutions containing NADH (250 μM) G6P (0.6 mM), IMVs (1.3×10^9 vesicles per mL) and G6PDH (0 (red) or 0.6 (green) U mL^{-1}). Control without IMVs in black. **(b)** G6P/NAD^+ couple. NADH concentration versus time (t) of solutions containing NAD^+ (250 μM) G6P (0.6 mM), IMVs (1.3×10^9 vesicles per mL) and G6PDH (0 (black) or 0.6 (green) U mL^{-1}). Control without IMVs in red. **(c)** Combination of (a) and (b). Reactions are performed in NaOH-Tricine buffer (100 mM, pH 8.0) with $MgCl_2$ 5 mM. Error bars are defined as s.d. (N = 3).

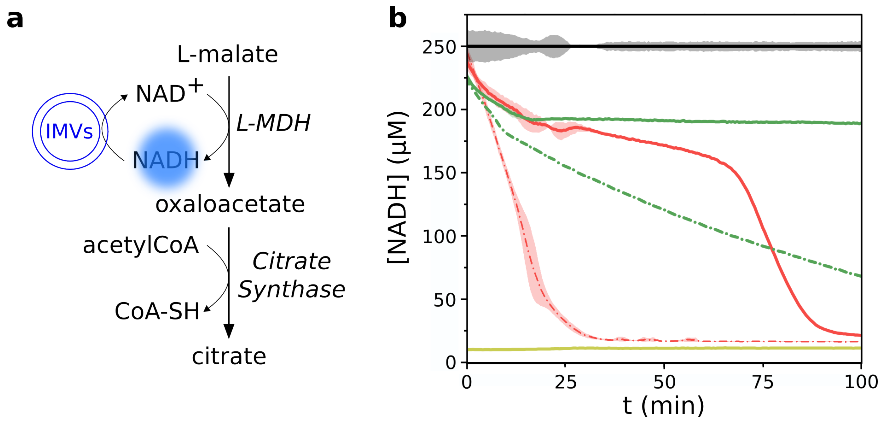

Supplementary Fig. 19: Self-sustained L-MDH activity. **(a)** L-malate dehydrogenase (L-MDH) activity (Supplementary Fig.9) is sustained when coupled to IMVs NADH oxidation activity. **(b)** NADH concentration versus time (t) of solutions containing NADH (250 μM), acetylCoA (0.4 mM), citrate synthase (10 U mL^{-1}), L-MDH (5 U mL^{-1}), L-malate (0 (dashed line) or 6 mM (straight line)) and IMVs (2×10^9 (red) or 4×10^8 vesicles per mL (green)). NADH (250 μM) and NAD$^+$ (250 μM) controls are shown in black and yellow respectively. Reactions are performed in KOH-Tricine buffer (100 mM, pH 8.0) with $MgCl_2$ 5 mM. Error bars are defined as s.d. (N = 3).

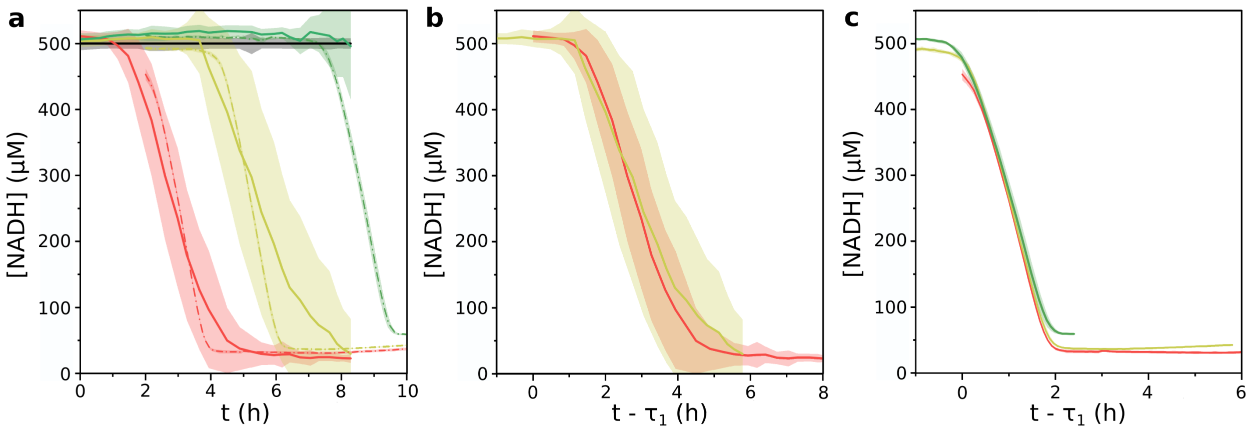

Supplementary Fig. 20: Self-sustained microcompartements. **(a)** NADH concentration versus time (t) of 30 pL w/o droplets containing NADH (500 μM), G6PDH (0.5 U mL^{-1}), IMVs (40 vesicles per droplet) and G6P (0.5 mM (red), 1 mM (yellow) or 2 mM (green)). NADH reference (500 μM) is shown in black. Error bars are defined as s.d. (N = 10 000). Dashed lines correspond to the respective reactions performed in 384-well plate (50 μl, N = 3). **(b)** Collapsing of the droplets data when the G6P 1 mM curve is shifted by time-scale τ_1 (τ_1 = 2.5 h). **(c)** Collapsing of the 384-well plate data when the G6P 1 mM curve is shifted by time-scale τ_1 (τ_1 = 2.2 h). The G6P 2 mM curve is also collapsing when shifted by τ_1 = 5.6 h. Reactions are performed in NaOH-Tricine buffer (100 mM, pH 8.0) with $MgCl_2$ 5 mM.

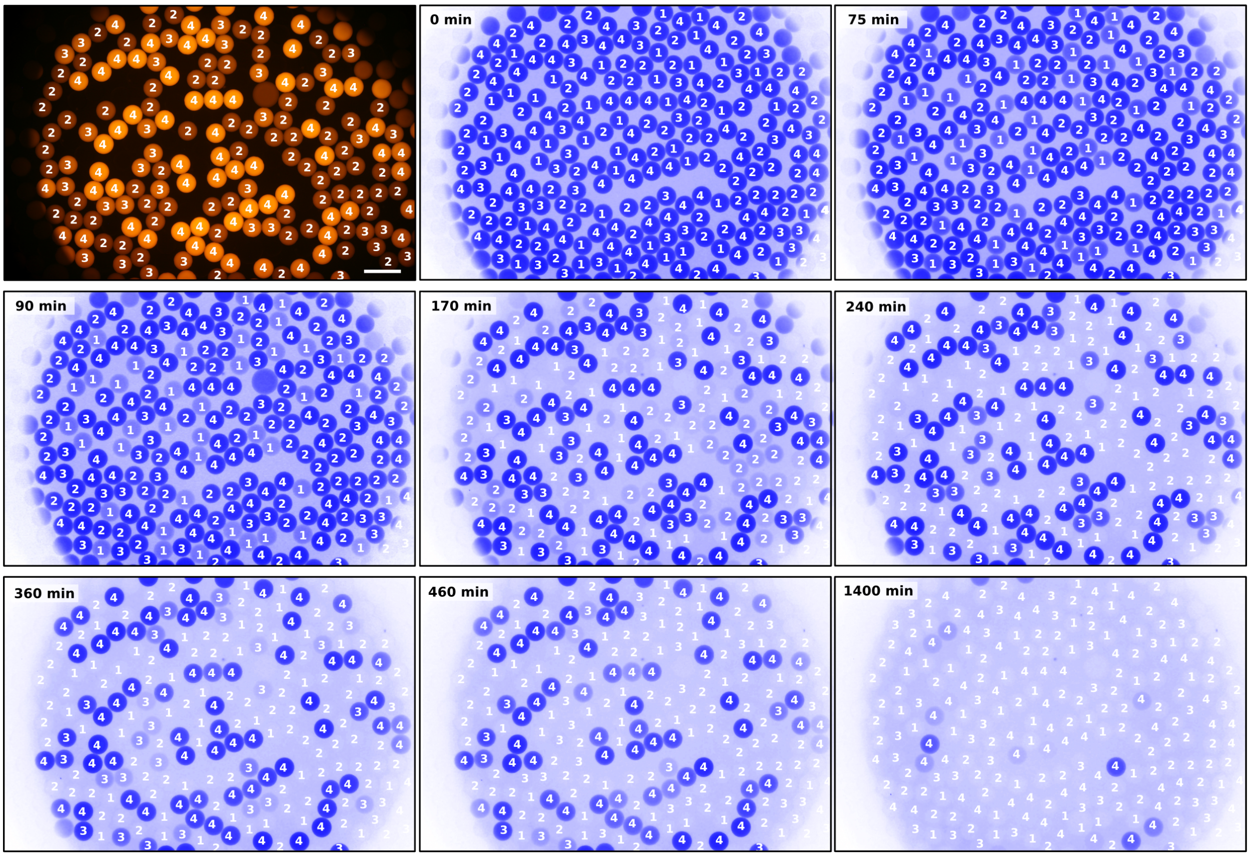

Supplementary Fig. 21: Time-lapse microscopy of self-sustained microcompartments. Red and blue fluorescence micrographs of 300 pL droplets containing NADH (1 mM), IMVs (1 200 vesicles per droplet), G6PDH (1 U mL^{-1}), sulforhodamine B (0, 5, 10 or 20 μM) and G6P (0.5, 1, 1.5 or 2 mM) during 23 h incubation. The metabolic activity of microcomparmtents decays over different times in the population (population 1 then population 2 then population 3 then population 4), as a function of G6P initial concentration, the more concentrated compartments being active the longest. Scale bar 200 μm. Reactions are performed in NaOH-Tricine buffer (100 mM, pH 8.0) with $MgCl_2$ 5 mM.

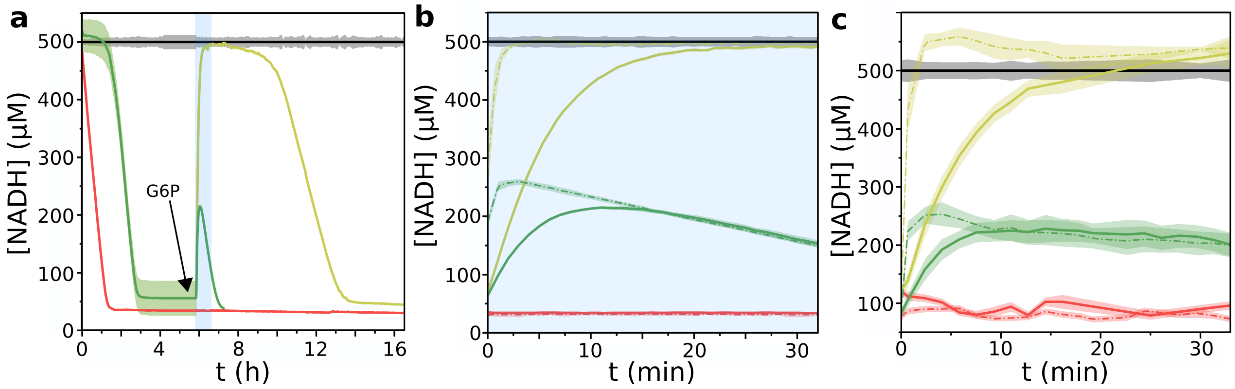

Supplementary Fig. 22: Substrate uptake. **a-b** Bulk kinetics. **(a)** NADH concentration versus time (t) of solutions containing NADH (500 μM), IMVs (7.2×10^8 vesicles per mL), G6PDH (0 (red) or 0.1 (green and yellow) U mL^{-1}) and G6P (0.5 mM). Control solution without IMVs in black. At t = 6h, addition of 0.2 mM (green) or 2 mM (red, yellow and black) G6P. Error bars are defined as s.d. (N = 3). **(b)** NADH concentration versus time (t) of solutions containing NADH (500 μM), IMVs (7.2×10^8 vesicles per mL), G6PDH (0 (red) or 0.1 (green and yellow) U mL^{-1}) and G6P (0.5 mM), after 6 h incubation and addition of 0.2 mM (green) or 2 mM (red, yellow and black) G6P. Control solution without IMVs in black. The dashed lines correspond to the same conditions but with 10-fold more G6PDH in the system (1 U mL^{-1}). Error bars are defined as s.d. (N = 3). **(c)** Droplets kinetics. NADH concentration versus time (t) of 30 pL droplets containing NADH (500 μM), IMVs (20 vesicles per droplet), G6PDH (0 (red) or 0.1 (green and yellow) U mL^{-1}) and G6P (0.5 mM), after 6 h incubation and picoinjection of 0.2 mM (green) or 2 mM (red, yellow and black) G6P. Control solution without IMVs in black. The dashed lines correspond to the same conditions but with 10-fold more G6PDH in the system (1 U mL^{-1}). Error bars are defined as s.d. (N = 5 000). Reactions are performed in NaOH-Tricine buffer (100 mM, pH 8.0) with $MgCl_2$ 5 mM.

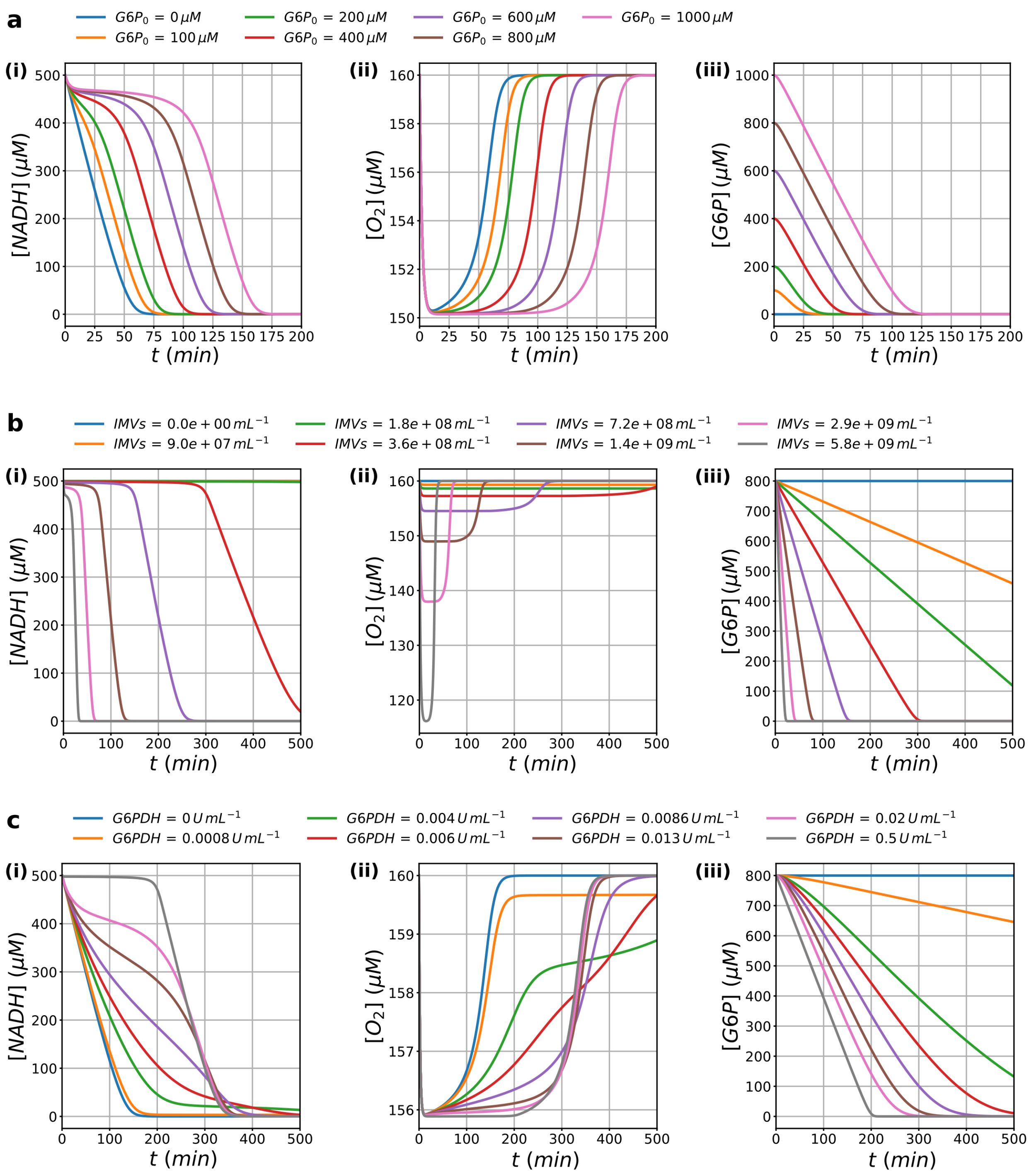

Supplementary Fig. 23: Numerical modelling of the reaction kinetics. The conditions and details of the numerics are provided in the Supplementary Note 6. Concentrations of NADH (i), Oxygen (ii) and substrate (iii) for varying initial substrate concentrations (a), for various IMV concentrations (b) and for various enzyme concentration (c). The graphs (i) reproduce the experimental results of Figure 3 (b,c,d) of the main text. The oxygen profile depends on the rate of reactions but also on the external supply (Supplementary Note 3). For reasonable values of the rate of transport from an external reservoir, the oxygen concentration remains close to the initial concentration.

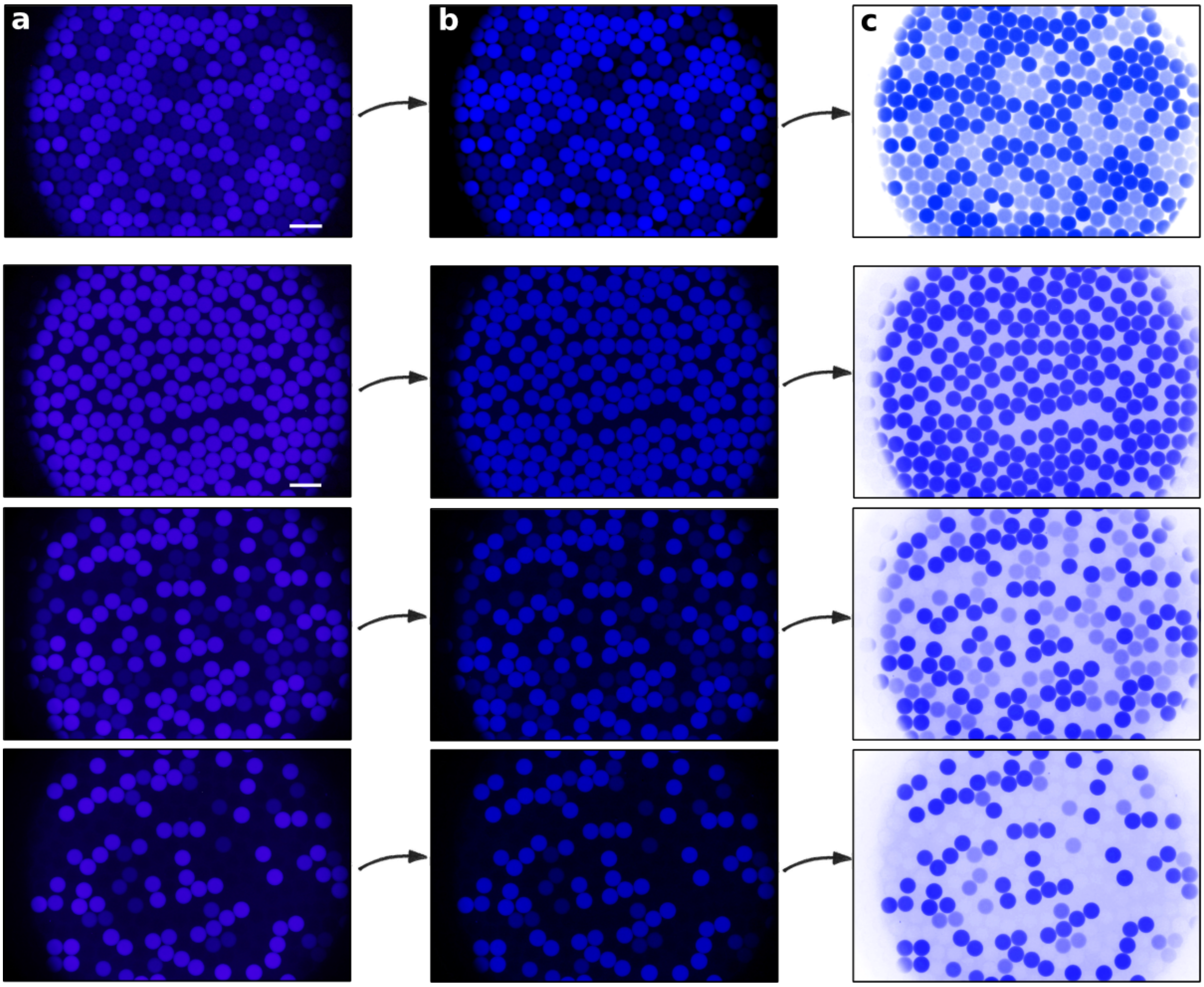

Supplementary Fig. 24: Image processing for droplets in the 2-d incubation chamber: (a) raw image; (b) after step 1; (c) after step 2. Details of the steps are detailled in Supplementary Note 5. Scale bars are 200 μm

Supplementary Note 1: Calibration of fluorescence measurements

A calibration curve was plotted for each fluorophore used as a read out. Using the drop-maker device described in Supplementary Fig. 1, a 8-bit emulsion was sequentially generated using 8 different fluorophore concentrations barcoded with a 8-bit sulforhodamine B coding. The 8-bit emulsion was analyzed in the simple reinjection device (Supplementary Fig. 2) to measure the fluorescence distribution of each 8 populations. Calibration curves were done for both fluorescein and NADH and were repeated in bulk in 384-well microtiter plates using a fluorospectrometer (Supplementary Fig. 6). All further experiments were performed within the linear concentration range with an internal standard (known concentration of a given fluorophore). Fluorescence intensities were translated into concentrations taking the internal standard as a reference.

Supplementary Note 2: Purification of Inverted Membrane Vesicles

E. coli (MG1655) were grown in LB medium containing 20 mM glucose. The cells were harvested in three centrifugation steps. First they were centrifuged for 20 minutes at 10 000 ×g at 4°C, the cells were resuspended in washing buffer (50 mM Tris-HCl pH 8.0, 1 mM EGTA) and centrifuged again for 20 min at 10.000 ×g at 4°C. The pellet was resuspended in washing buffer and centrifuged a third time for 30 min at 5 000 ×g at 4°C. Afterwards the pellet was frozen in liquid nitrogen and stored at −80°C.

The cell pellet was covered with washing buffer, thawed in a water bath at 30°C and then resuspended. The suspension was centrifuged for 10 min at 15 000 ×g at 4°C and the resulting pellet was resuspended in 300 mL washing buffer. The cells were centrifuged again for 10 min at 15.000 ×g at 4°C and the pellet was resuspended in lysis buffer (50 mM MOPS, 175 mM KCl, 10 mM $MgCl_2$, 0.2 mM EGTA, 0.2 mM DTT, 0.1 mM PMSF, pH 7.0), homogenized with a glass tissue homogenizer or stirred on ice for 1 hour. 1 unit per g cell mass of DNAse was added and the cells were pressed through a cooled French press three times at 1000 bar. Alternatively, ultrasonication was used for IMVs preparation. In that case, the cells were lysed for a duration of 3 min with 0.5 second pulses, 5 sec pauses and a 65% amplitude. The solution was examined with a microscope to determine whether the French press treatment was successful. The homogenized cells were frozen in liquid nitrogen and stored overnight at −80°C.

The frozen suspension was thawed in a water bath at room temperature and centrifuged for 20 min at 25 000 ×g at 4°C and the pellet was discarded. The membrane was isolated by ultracentrifugation according to [1]. The supernatant was ultracentrifuged (Ultrazentrifuge Optima XPN 100, Beckman Coulter) for 120 min at 433 000 ×g at 4°C. After this step the pellet contained the inner and outer membrane and was resuspended in membrane buffer (50 mM Tris-HCl, 0.2 mM EGTA, 5 mM $MgCl_2$, 6 mM PAB, 10 % (v/v) glycerin, 2 mM DTT, 0.1 mM PMSF, pH 8.0) using a brush. The resuspended pellet was ultacentrifuged again for 90 min at 433 000 ×g at 4°C and resuspended in membrane buffer. The last ultracentrifugation step took 90 min at 433 000 ×g at 4°C and the pellet was resuspended in membrane buffer. The solution was frozen in liquid nitrogen and stored overnight at −80°C and next day thawed again in a water bath at 30°C. Next, a density gradient centrifugation was performed [2, 3]. The ultracentrifugation tubes were filled with 6 mL 50 % sucrose, 8

mL 40 % sucrose, 10 mL 30 % sucrose, 10 mL 20 % sucrose and 1-2 mL sample on top. The tubes were centrifuged for 24 hours at 240 000 ×g (swinging bucket rotor SW 32 Ti) with acceleration and deceleration at the lowest possible level. The membrane was located between 35 % and 45 % sucrose and the darker fraction was carefully collected. The protein content of the fractions was measured with a nanodrop and they were resuspended 1:4 in membrane buffer. The resulting solution was ultracentrifuged for 2 hours at 433 000 ×g (fixed angle rotor type 70 Ti) and the pellet was dried and weighted [4]. The pellet was resuspended in membrane buffer to a concentration of 2 g pellet per mL buffer and the solution was pressed through a sterile filter (0.22 μm). The resulting solution containing the IMVs was finally frozen in liquid nitrogen in 500 μL aliquots and stored at -80°C [5].

Supplementary Note 3: NADH-driven ATP production and oxygen consumption of IMVs

The measurements of NADH-driven ATP production in IMVs were performed in a microplate reader (Biotech, Synergy HT) using the luciferin-luciferase assay. To a solution containing 50 μL of measurement buffer (20 mM Tris acetate (pH 7.8), 5 mM magnesium acetate, 1 mM DTT, 0.5 mM KH2PO4, 0.25 mM $MgCl_2$, 0.25 mM ADP, 0.1 mM EDTA) and 50 μL luciferin/luciferase assay (CLSII, prepared double concentrated according to the manufacturer's protocol), 2.5 μL IMVs were added (the stock solution contained 2.2×10^{11} vesicles per mL) and the baseline was recorded. The reaction was initiated by the addition of 10 μL NADH (2 mM, 1.5 mM, 1 mM, 0.5 mM, 0.2 mM and 0.1 mM stock solutions) as soon as a constant background was detected (Supplementary Fig. 12a). The initial rates of NADH oxidation were determined in the same experimental setup (Supplementary Fig. 12b).

The oxygen consumption of IMVs was determined using an oxygraph equipped with a Clark-type electrode (Oxytherm, Hansatech) (Supplementary Fig. 12c). To a solution containing 450 μL buffer (100 mM NaOH-Tricine (pH 8.0), 5 mM $MgCl_2$), 10 μL IMVs stock solution was added and the reaction was initiated upon addition of 40 μL NADH stock solutions, such as the final NADH concentrations were 0, 0.25, 0.5 and 1 mM. Thus, final IMVs concentration was 4.4×10^9 vesicles per mL, corresponding to 50$\times$ dilution. In addition, another experiment with 100$\times$ dilution (2.2×10^9 vesicles per mL) and 0.5 mM final NADH concentration was performed to probe the influence of IMVs concentration on the oxygen consumption rate. The initial rate was independent on the NADH concentration (0.25, 0.5 and 1 mM) but decreased twice when the IMVs concentration was doubly reduced in accordance with the linear dependence with respect to NADH (Supplementary Fig. 13c). In the case of 0.5 mM NADH, which was further used in the microfluidic measurements, the oxygen concentration decreased with about 20%. After consumption of the NADH the oxygen concentration was equilibrated to the initial values due to the fact that the measurement cuvette was open to the ambient air (Supplementary Fig. 12c).

Supplementary Note 4: 2D observatory chamber assembly

A 2D observation chamber was assembled adapting the protocol described in [6] (Supplementary Fig. 15). Glass microscopy slides were used as top and bottom covers (76 x 25 x 1 mm, Marienfeld). Two access holes of 1.5 mm diameter were created in the top glass slide using micro-sandblasting. Both slides were thoroughly cleaned using soap, water, ethanol and acetone and dried at 70°C. The chamber geometry was cut in a 60 μm-thick double-sided bonding tape (1375, SDAG Adhésifs) using a Graphtech cutting plotter (CE 6000-40). The double-sided bonding template was transferred on the bottom glass slide and the top glass slide was then bonded to seal the system. The chamber was incubated 24 h at 70°C. Next, two nanoports were attached to the holes using a UV curable glue (Loctite 3526, Henkel). Subsequently, the surface of the 2D chamber was treated using fluoro-silane (Aquapel, Aquapel). Lastly, the chamber was dried under argon, filled with fluorinated oil and sealed until used. The chamber was reused multiple times and cleaned after each experiment by flushing fluorinated oil. This procedure resulted in a chamber having the following dimensions : 35 mm x 10 mm x 60 μm.

Supplementary Note 5: Post-treatment of blue fluorescence images

NADH fluorescence images were taken with a digital camera (Canon, EOS D600) using the epifluorescence setup described in Methods. For better clarity, raw images were slighlty processed using the following post-treatment using ImageJ 1.51k sofware: first, red channel was removed from raw images (step 1). Then, colors were inverted and the hue of the yellow color was maximized (step 2). Supplementary Figure 24 gives examples of images at each step of the process.

Supplementary Note 6: Modelling kinetics

We consider the coupled reaction as described in Figure 3a, Main Text:

$$NAD^+ + G6P \xrightarrow{G6PDH} GLP + NADH + H^+ \tag{1}$$

$$NADH + H^+ + \frac{1}{2}O_2 \xrightarrow{IMV} NAD^+ + H_2O \tag{2}$$

Glucose-6-phosphate dehydrogenase is a model enzyme for studies on bisubstrate reactions and the protein isolated from *Leuconostoc mesenteroides*, which can utilize NADH alongside NAD(P)H with comparable rates, has been characterized in detail. Levy and coworkers assigned ordered sequential mechanism with NADH and speculated about isomerization of enzyme [7] but afterwards revised the kinetics to random mechanism based on additional experiments [8]. Since ordered and random mechanisms are indistinguishable with respect to initial velocity expressions [9] and high mechanistic precision would not affect the outcome of the model, we assigned ordered mechanism as in [7] (Eq. 4 in Supplementary Note) and used the available Michaelis and inhibition (complex dissociation) constants. Regarding the IMVs, the approaches for quantitative formulation of the electron transport chain and oxidative phosphorylation kinetics generally range from non-equilibrium thermodynamics [10] to kinetic models at various approximation levels [11]. The aerobic electron transport chain of E. coli comprises different NADH dehydrogenases and terminal oxidases linked through a quinone pool [12] and in some cases the respiratory complexes have been modeled through irreversible kinetics of Michaelis-Menten type [13]. Provided that we did not have information about the exact protein compostion of the IMVs and did not have sufficient experimental evidences to assign complex kinetics, we arbitrarily considered the IMVs as one enzyme complex, which oxidizes NADH and reduces oxygen and sought for the simplest kinetic expression that would describe the observed experimental behavior in terms of NADH oxidation functionality. The NADH and oxygen reaction rates depended linearly on IMVs concentration (Figure S11b, S12s, S13c) but there was little dependence on the NADH concentration in the higher range (>250 μM, Figure S11a), which was used in microfluidic experiments. Therefore, based on the assumption that the level of oxygen was sufficiently high as discussed above, we assigned kinetics of Michaelis-Menten type, whereby the rate constant k_2 was defined at the given oxygen concentration. The oxygen transfer rate (OTR) was conventionally expressed (Eq. 8) through the constant k_La, whereby the

saturation concentration was taken from oxygen measurements (Figure S11b). The fitted mass transfer coefficient $k_L a$ was rescaled to be consistent with the well plate experiments in Figure 3 through the surface-to-volume ratio a. These assumptions led to the mathematical description:

$$r_1 = \frac{a_1 \cdot [G6P] \cdot [NAD^+]}{K_i^{NAD^+} K_m^{G6P} + K_m^{G6P}[NAD^+] + K_m^{NAD^+}[G6P] + [G6P][NAD^+]} \tag{3}$$

$$a_1 = k_{cat}^{G6PDH} \cdot [G6PDH] \tag{4}$$

$$r_2 = \frac{a_2 \cdot [NADH]}{K_m + [NADH]} \tag{5}$$

$$a_2 = k_2([O_2]) \cdot \frac{[IMV_0]}{N_A} \cdot 10^9 \tag{6}$$

$$OTR = k_L a([O_2]^* - [O_2]) \tag{7}$$

and:

$$\frac{d[G6P]}{dt} = -r_1 \tag{8}$$

$$\frac{d[GLP]}{dt} = r_1 \tag{9}$$

$$\frac{d[NAD^+]}{dt} = -r_1 + r_2 \tag{10}$$

$$\frac{d[NADH]}{dt} = r_1 - r_2 \tag{11}$$

$$\frac{d[O_2]}{dt} = OTR - 0.5 r_2 \tag{12}$$

We used the initial conditions detailed in Supplementary Table 1. A parameter set was estimated (Supplementary Table 2) to fit the experimental datasets of different experiments.

Supplementary Table 1: Initial Conditions

$NADH_{tot}$	500 μM
$G6P$	0-1000 μM
$G6PDH$	0-0.5 $\frac{U}{mL}$
O_2	160 μM
O_2^*	160 μM
IMV_0	0-5.8e9 $\frac{vesicles}{mL}$

The parameter estimation was performed by minimizing the residual sum of squares RSS between simulation and experimental datasets, using the toolbox Copasi [14]. The optimization algorithm evolutionary programming was used to identify an approximation of a parameter set for a suitable global minimum of the RSS [15]. Additionally, the gradient orientated simplex algorithm was applied to certainly reduce the RSS into potential global minimum and optimal parameter set, respectively [16]. Since the RSS around the global minimum quickly exceed the confidence limits, characterized by a F-distribution with 95 percent upper α-critical value, n constants and m data points of measurement

$$RSS(p) \leq RSS(p^*) \left(1 + \frac{n}{m-n} F^{95\%}_{n,m-n}\right), \tag{13}$$

the fitted kinetic constants (p) are identifiable and can be considered as reliable in the respect confidence interval [17].

Supplementary Table 2: Parameter Set

Parameter	Fitted Value	Source
$k_L a$ cuvette	$0.006\ s^{-1}$	fitted with cuvette experiments ($a = 567 \mu m^{-1}$)
$k_L a$ well plate	$0.008\ s^{-1}$	$a = 831 \mu m^{-1}$
k_{cat}^{G6PDH}	$9.7\ \mu M \cdot mL \cdot U^{-1} \cdot s^{-1} \pm 0.7$	fitted with well plate experiments
$K_m^{G_6P}$	$52.7\ \mu M$	[7]
$K_m^{NAD^+}$	$106\ \mu M$	[7]
$K_i^{NAD^+}$	$763\ \mu M$	[7]
k_2	$82500\ s^{-1} \pm 2600$	fitted with well plate experiments
$K_{m,2}$	$41\ \mu M \pm 12$	fitted with well plate experiments

Supplementary References

[1] Heitkamp, T. *et al.* Monitoring subunit rotation in single fret-labeled fof1-atp synthase in an anti-brownian electrokinetic trap. *SPIE Bios. International Society for Optics and Photonics* (2013).

[2] Miura, T. & Mizushima, S. Separation by density gradient centrifugation of two types of membranes from spheroplast membrane of escherichia coli k12. *Biochimica et Biophysica Acta* **150**, 159–161 (1968).

[3] Jewett, M. C., Calhoun, K. A., Voloshin, A., Wuu, J. J. & Swartz, J. R. An integrated cell-free metabolic platform for protein production and synthetic biology. *Molecular Systems Biology* **4**, 220 (2008).

[4] Osborn, M., Gander, J., Parisi, E. & Carson, J. Mechanism of assembly of the outer membrane of salmonella typhimurium isolation and characterization of cytoplasmic and outer membrane. *Journal of Biological Chemistry* **247**, 3962–3972 (1972).

[5] Wuu, J. & Swartz, J. R. High yield cell-free production of integral membrane proteins without refolding or detergents. *Biochimica et Biophysisca Acta* **1778**, 1237–1250 (2008).

[6] Eyer, K. *et al.* Single-cell deep phenotyping of IgG-secreting cells for high-resolution immune monitoring. *Nature Biotechnology* **35**, 977–982 (2017).

[7] Olive, C., Geroch, M. E. & Levy, H. R. Glucose 6-phosphate dehydrogenase from Leuconostoc mesenteroides. Kinetic studies. *The Journal of biological chemistry* **246**, 2047–57 (1971).

[8] Levy, H. R., Christoff, M., Ingulli, J. & Ho, E. M. Glucose-6-phosphate dehydrogenase from Leuconostoc mesenteroides: Revised kinetic mechanism and kinetics of ATP inhibition. *Archives of Biochemistry and Biophysics* **222**, 473–488 (1983).

[9] Frieden, C. On the kinetic distinction of ordered and random bireactant enzyme systems. *Biochemical and Biophysical Research Communications* **68**, 914–917 (1976).

[10] Jin, Q. & Bethke, C. M. Kinetics of electron transfer through the respiratory chain. *Biophysical Journal* **83**, 1797–1808 (2002).

[11] Korzeniewski, B. & Zoladz, J. A. A model of oxidative phosphorylation in mammalian skeletal muscle. *Biophysical Chemistry* **92**, 17–34 (2001).

[12] Unden, G. & Bongaerts, J. Alternative respiratory pathways of Escherichia coli: Energetics and transcriptional regulation in response to electron acceptors (1997).

[13] Henkel, S. G. *et al.* Basic regulatory principles of Escherichia coli's electron transport chain for varying oxygen conditions. *PLoS ONE* **9**, e107640 (2014).

[14] Hoops, S. *et al.* COPASI - A COmplex PAthway SImulator. *Bioinformatics* **22**, 3067–3074 (2006).

[15] Bäck, T. & Schwefel, H.-P. An Overview of Evolutionary Algorithms for Parameter Optimization. *Evolutionary Computation* **1**, 1–23 (1993).

[16] Nelder, J. A. & Mead, R. A Simplex Method for Function Minimization. *The Computer Journal* **7**, 308–313 (1965).

[17] Schaber, J. Easy parameter identifiability analysis with COPASI. *Biosystems* **110**, 183–185 (2012).
