# Supplementary material for: Out-of-equilibrium microcompartments for the bottom-up integration of metabolic functions
Source: Nat Commun. 2018 Jun 19;9:2391. doi: 10.1038/s41467-018-04825-1 (PMC6008305; doi:10.1038/s41467-018-04825-1)
Supplement: Supplementary file 3 — Description of Additional Supplementary Files [file 41467_2018_4825_MOESM3_ESM.pdf]

Description of Additional Supplementary Files

File Name: Supplementary Movie 1
Description: Time-lapse microscopy of self-sustained metabolically active microcompartments. The 300 pL droplets emulsion is composed of 4 populations fed with 4 different G6P substrate concentrations.

File Name: Supplementary Movie 2
Description: Time-lapse microscopy of self-sustained metabolically active microcompartments. The 300 pL droplets emulsion is composed of 4 populations fed with 4 different G6P substrate concentrations.
